# Supplementary material for: Identification of photoperiod-induced specific miRNAs in the adrenal glands of Sunite sheep (Ovis aries)
Source: Front Vet Sci. 2022 Jul 22;9:888207. doi: 10.3389/fvets.2022.888207 (PMC9354845; doi:10.3389/fvets.2022.888207)
Supplement: Supplementary file 1 [file Data_Sheet_1.ZIP › Supplementary file/miRDeep2 file.zip!/miRDeep2 file/Novel_Structure/SP42A2/Novel_698.pdf]

Provisional ID	:	NC_019483.2_41935
Score total	:	25.8
Score for star read(s)	:	3.9
Score for read counts	:	18
Score for mfe	:	2.3
Score for randfold	:	1.6
Score for cons. seed	:	
Total read count	:	47
Mature read count	:	46
Loop read count	:	0
Star read count	:	1

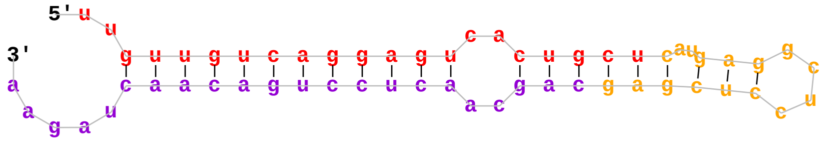

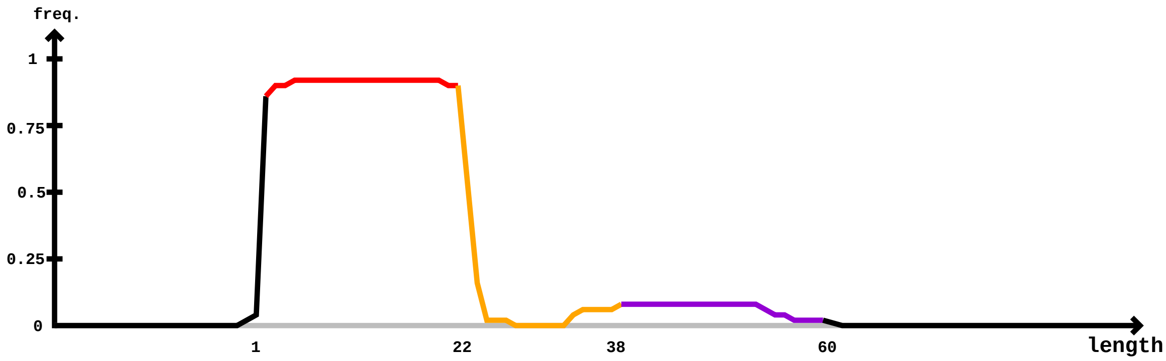

Mature **Star**

```
5'- gacgauguaggcucacccacuuguugucaggagucacugcucaugaggcuccucgagcagcaacuccugacaacuagaagggagggcagagcaucagcgcagccccugugg   -3'   obs
    gacgauguaggcucacccacuuguugucaggagucacugcucaugaggcuccucgagcagcaacuccugacaacuagaagggagggcagagcaucagcgcagccccugugg         exp
    ...(((((..((((.(((.((.((((((((((((..((((((..(((....)))))))))..)))))))))))).))..))).))))...)))))..(((((...))))).    reads   mm   sample
    ..................acuuguugucaggagucacug........................................................................    1       0    seq
    ...................cuuguugucaggagucacugcu......................................................................    1       0    seq
    ....................uuguugucaggagucacugcu......................................................................    19      0    seq
    ....................uuguugucaggagucacugcuc.....................................................................    16      0    seq
    ....................uuguugucaggagucacugcuca....................................................................    6       0    seq
    .....................uguugucaggagucacugcuc.....................................................................    1       0    seq
    .....................uguugucaggagucacugcuca....................................................................    1       0    seq
    .......................uugucaggagucacugcucauga.................................................................    1       0    seq
    ....................................................ucgagcagcaacuccugaca.......................................    1       0    seq
    ....................................................ucgagcagcaacuccugacaa......................................    1       0    seq
    .....................................................cgagcagcaacuccugacaacu....................................    1       0    seq
    .........................................................cagcaacuccugacaacuagaa................................    1       0    seq
```
